# L'observance médicamenteuse et ses facteurs dans un groupe d'hypertendus congolais

**DOI:** 10.11604/pamj.2013.15.121.2435

**Published:** 2013-08-06

**Authors:** Méo Stéphane Ikama, Bernice Mesmer Nsitou, Mpouoni Loumouamou, Gisèle Kimbally-Kaky, Jean Louis Nkoua

**Affiliations:** 1 Service de Cardiologie, CHU de Brazzaville, République du Congo

**Keywords:** Observance médicamenteuse, hypertension artérielle, Congo, drug compliance, hypertension, Congo

## Abstract

L'objectif de ce travail était d’évaluer le niveau d'observance des patients hypertendus et identifier les facteurs prédictifs de mauvaise observance. Il s'est agi d'une étude transversale à recueil de données prospectif sur une période de six mois. Elle s'est déroulée dans les services de consultations externes de cardiologie dans trois hôpitaux: le CHU de Brazzaville, l'hôpital central des armées de Brazzaville, et l'hôpital général de Loandjili (Pointe-Noire). Cette étude a concerné 212 patients dont 122 femmes (57.5%) et 90 hommes (42.5%), âgés de plus de 18ans, hypertendus traités depuis au moins six mois. Le questionnaire à six questions conçu par Girerd a été utilisé pour évaluer l'observance médicamenteuse. Une régression logistique a été utilisée pour rechercher les facteurs de mauvaise observance. L'observance était considérée comme bonne chez 45 patients soit 21.2%, et mauvaise chez 69 patients soit 32.5%. Dans 98 cas soit 46.2% il y avait un minime problème d'observance. Une HTA ancienne de plus de 5 ans, la présence des complications évolutives de l'HTA ainsi que les niveaux élevés de la PAS et de la PAD étaient liés à la mauvaise observance. Par contre aucune relation statistique n'a été retrouvée entre la mauvaise observance et l'existence d'une co-morbidité, la fréquence des prises médicamenteuses, le nombre de comprimés par jour et une pression artérielle non contrôlée. Les patients non observant étaient en moyenne plus âgés que les autres. Une mauvaise connaissance du traitement et des complications de l'HTA, le fait de ne pas acheter soi-même ses médicaments, l'ignorance de la gravité de l'HTA, et le coût élevé du traitement étaient prédictifs d'une mauvaise observance. La possession d'un tensiomètre électronique d'auto mesure tensionnelle ainsi que le rappel des prises médicamenteuses par les parents étaient liés à une bonne observance. Après ajustement par une régression logistique, seule la bonne connaissance du traitement et la perception de la gravité de l'HTA étaient liées à une bonne observance. L'observance médicamenteuse dans notre population d’étude s'est révélée faible. Il faut insister sur l’éducation thérapeutique des patients.

## Introduction

Des progrès considérables ont été réalisés sur le traitement de l'hypertension artérielle (HTA), et le bénéfice du traitement sur le contrôle tensionnel et la réduction de la morbi-mortalité ne sont plus à démontrer. Pourtant, sur l'ensemble des patients traités, moins de la moitié seulement ont une HTA contrôlée (44.9% en France) [1]. Parmi les causes de non contrôle de la tension artérielle figure en bonne place l'observance insuffisante du traitement [2, 3].

De nombreux travaux cliniques et épidémiologiques se sont efforcés de mesurer le degré d'observance des hypertendus traités. Ce degré est très variable dans la littérature en fonction des populations [4, 5].

Au Congo, l'HTA constitue un véritable problème de santé publique avec une prévalence estimée à 32.5% à Brazzaville [6]. Cependant, nous ne disposons pas de données quantitatives sur le degré d'observance des patients traités. La présente étude, visant à réduire la morbi-mortalité chez les patients hypertendus congolais, s'est fixée pour objectifs: d’évaluer le niveau d'observance chez les patients hypertendus; d'identifier les facteurs prédictifs de mauvaise observance.

## Méthodes

Il s'est agi d'une étude transversale à recueil prospectif sur une période de six mois, allant de décembre 2010 à juin 2011. Elle s'est réalisée dans les unités de consultations externes de cardiologie de trois hôpitaux du Congo (Centre Hospitalier Universitaire de Brazzaville, Hôpital Central des Armées de Brazzaville, Hôpital Général de Loandjili de Pointe-Noire). Elle a inclus des patients âgés de 18 ans et plus, suivis et traités pour une HTA depuis au moins six mois. Les patientes souffrant d'une HTA gravidique ont été exclues. Pour évaluer l'observance, nous avons utilisé le test mis au point et validé par GIRERD et al. [5], comportant six questions auxquelles le patient doit répondre par oui ou non (Tableau 1). Pour étudier les facteurs prédictifs de mauvaise observance, les patients ont été répartis en deux groupes: les mauvais observants (total des oui ≥3) et les bons observants (total des oui < 3). Les patients ayant de minimes problèmes d'observance ont été classés dans le groupe des bons observants.

**Tableau 1:** Test d’évaluation de l'observance [4]

Test d’évaluation de l'observance	Oui	Non
1. Ce matin avez-vous oublié de prendre votre médicament ?		
2. Depuis la dernière consultation, avez-vous été en panne de médicament ?		
3. Vous est-il arrivé de prendre votre traitement avec retard par rapport à l'heure habituelle ?		
4. Vous est-il arrivé de ne pas prendre votre traitement parce que certains jours, votre mémoire vous fait défaut ?		
5. Vous est-il arrivé de ne pas prendre votre traitement parce que certains jours, vous avez l'impression que votre traitement vous fait plus de mal que de bien ?		
6. Pensez-vous que vous avez trop de comprimés à prendre ?		
Total des OUI:		
**Interprétation du test** Total des OUI = 0 Bonne observance Total des OUI = 1 ou 2 Minime problème d'observance Total des OUI ≥ 3 Mauvaise observance		

Plusieurs paramètres ont été étudiés, répartis en paramètres sociodémographiques (âge, sexe, profession, niveau d'instruction, statut matrimonial), cliniques et thérapeutiques (ancienneté de l'HTA, présence d'une complication de l'HTA ou d'une co-morbidité, connaissance du patient sur son traitement, classes thérapeutiques et protocoles utilisés, nombre de comprimés et nombre de prises par jour).

L'HTA était considérée comme contrôlée pour des chiffres inférieurs à 140/90 mmHg dans la population générale, et inférieurs à 130/80 mmHg chez les diabétiques ou les insuffisants rénaux [7], sur une moyenne de trois mesures.

Les données ont été analysées par les logiciels Epi-Info 3.5.1 et SPSS 11.1. Pour les comparaisons, nous avons utilisé le test Khi-2 pour les variables qualitatives et l'analyse des variances (ANOVA) pour les variables quantitatives. La recherche des facteurs de mauvaise observance s'est faite à l'aide d'une régression logistique. Le seuil de significativité a été fixé à p < 0,05.

## Résultats

Deux cents douze patients hypertendus ont été retenus, d’âge moyen de 58,3 ± 10,6 ans (extrêmes: 34 et 81 ans). Il s'agissait de 122 femmes (57.5%) et 90 hommes (42.5%), sans différence d’âge (p = 0.3).

Les principales caractéristiques de la population sont consignées dans le Tableau 2. L'HTA était ancienne en moyenne de 6.4 ± 5.7 ans (extrêmes: 1 et 25 ans). Les moyennes de PAS et de PAD étaient respectivement de 146.5 ± 22.7 mmHg et 87.6 ± 13.6 mmHg.

**Tableau 2:** Caractéristiques de la population d’étude

	n (%)
Sexe féminin	122 (57,5)
Age moyen (ans)	58,3 ± 10,6
Mariés (vivant en couple)	140 (66,0)
Sans niveau d'instruction	48 (22,6)
Sans profession	63 (29,7)
Ancienneté de l'HTA (ans)	6,4 ± 5,7
Niveau de contrôle tensionnel	56 (26,4)
Monothérapie	45 (21,2)
Polythérapie	167 (78,8)
Bonne observance	45 (21,2)
Mauvaise observance	69 (32,5)
Minime problème d'observance	98 (46,2)

Seuls 56 patients (26.4%) sur les 212 traités étaient à l'objectif tensionnel. Les protocoles thérapeutiques utilisés étaient une monothérapie (35.4%), une bithérapie (47.6%), une trithérapie (12.3%), et une quadrithérapie (4.7%).

Le nombre moyen de comprimés pris par jour était de 2.6 ± 1.9 (extrêmes: 1 et 9), avec en moyenne 1.5 ± 0.6 prise/jour (extrêmes: 1 et 3). L'observance était considérée comme bonne chez 45 patients (21.2%), et mauvaise chez 69 (32.5%). Dans 98 cas (46.2%), il y avait un minime problème d'observance. Les déterminants de l'observance médicamenteuse liés à l'HTA sont représentés dans le Tableau 3.

**Tableau 3:** Déterminants de l'observance liés à l'HTA

Variables explicatives	Observance		OR (IC 95%)	p
	Mauvaise	Bonne		
**Ancienneté de l'HTA**				
Inferieur à 5 ans	35 (50,7%)	93 (65,0%)	0,55(0,30-0,99)	0,04
Supérieure à 5 ans	34 (49,3%)	50 (35,0%)		
**Co-morbidité**				
Non	42 (60,9%)	102 (71,3%)	0,62 (0,34-1,14)	0,1
Oui	27 (39,1%)	41 (28,7%)		
Fréquence des prises (x±ET)	1,59±0,55	1,45±0,64		0,1
Nombre de comprimés par jour (x±ET)	2,60±1,77	2,62±2		0,9
**Complication de l'HTA**				
Non	23 (33,3%)	79 (55,2%)	0,40 (0,22-0,73)	0,002
Oui	46 (66,7%)	64 (44,8%)		
PAS	151,16 ±24	144,3 ±21		0,04
PAD	91,1± 12	85,9 ±14		0,008
**Contrôle tensionnel**				
Non	55 (79,7%)	101 (70,6%)	1,63(0,82-3,25)	0,15
Oui	14 (20,3%)	42 (29,4%)		

Une HTA ancienne de plus de 5 ans, la présence de complications évolutives de l'HTA ainsi que les niveaux élevés de PAS et de PAD étaient liés à la mauvaise observance.

Le Tableau 4 représente les déterminants de l'observance médicamenteuse liés au patient. Ainsi, les patients mauvais observants étaient en moyenne plus âgés que les autres. Une mauvaise connaissance du traitement et des complications de l'HTA, le fait de ne pas acheter soi-même ses médicaments, l'ignorance de la gravité de l'HTA, et le coût élevé du traitement étaient prédictifs d'une mauvaise observance.

**Tableau 4:** Déterminants de l'observance liés au patient

Variables explicatives	Observance		OR (IC 95%)	p
	Mauvaise	Bonne		
Age (x ± ET)	60.4±10.5	57.2±10.6		0.04
**Sexe**				
F	34 (49.3%)	88 (61.5%)	0.6 (0.34-1.08)	0.06
M	35 (50.7%)	55 (38.5%)		
**Etat civil**				
Célibataire	29 (42%)	43 (30.1%)	1.68 (0.93-3.06)	0.08
Marié	40 (58%)	100 (69.9%)		
**Profession**				
Travailleurs	43 (62.3%)	106 (74.1%)	0.69 (0.47-1.03)	0.07
Sans travail	26 (37.7%)	37 (25.9%)		
**Couverture sociale**				
Non	65 (94.2%)	131 (91.6%)	1.48 (0.46-4.8)	0.5
Oui	4 (5.8%)	12 (8.4%)		
**Connaissance du traitement**				
Non	39 (56.5%)	34 (23.8%)	4.16 (2.25-7.68)	< 10 -5
Oui	30 (43.5%)	109 (76.2%)		
**Connaissance des complications de l'HTA**				
Non	39 (56.5%)	44 (30.8%)	2.9 (1.61-5.29)	0.0003
Oui	30 (43.5%)	99 (69.2%)		
**Achat des médicaments**				
Autre personne	44 (63.8%)	64 (44.8%)	2.17 (1.20-3.92)	0.009
Malade seul	25 (36.2%)	79 (55.2%)		
**Perceptions et attitudes**				
**Efficacité du traitement**				
Non	4 (5.8%)	6 (4.2%)	1.4 (0.38-5.15)	0.6
Oui	65 (94.2%)	137 (95.8%)		
**Gravité de l'HTA**				
Non	18 (26.1%)	14 (9.8%)	3,25 (1.5-7.02)	0.001
Oui	51 (73.9%)	129 (90.2%)		
**Effets indésirables perçus**				
Non	63 (91.3%)	117 (81.8%)	2.33 (0.91-5.96)	0.7
Oui	6 (8.7%)	26 (18.2%)		
**Cout élevé du traitement**				
Non	28 (40.6%)	88 (61.5%)	0.42 (0.23-0.76)	0.004
Oui	41 (59.4%)	55 (38.5%)		
**Possession d'un appareil d'auto mesure**				
Non	60 (87%)	97 (67.8%)	3.16 (1.44-6.92)	0.002
Oui	9 (13%)	46 (32.2%)		
**Rappel des prises médicamenteuses par la famille**				
Oui	31 (44.9%)	87 (60.8%)	0.52 (0.29-0.93)	0.03
Non	38 (55.1%)	56 (39.2%)		

Après ajustement par une régression logistique, seule la bonne connaissance du traitement et la perception de la gravité de l'HTA favorisent une bonne observance (Tableau 5).

**Tableau 5:** Résultats de la régression logistique

	O R	I C 95%		Coefficient	p
Achat seul des médicaments	1.0173	0.4398	2.353	0.0172	0.968
AGE	0.9864	0.9496	1.0245	-0.0137	0.4783
Ancienneté de l'HTA	1.059	0.9945	1.1276	0.0573	0.0736
Possession d'un appareil d'auto mesure	0.5448	0.2115	1.4029	-0.6074	0.2082
Cout élevé du traitement	1.8414	0.9315	3.6401	0.6105	0.0791
Présence des complications	1.5848	0.7415	3.3872	0.4605	0.2347
Connaissance des complications de l'HTA	0.7675	0.3213	1.8332	-0.2646	0.5514
PAD	1.0252	0.9929	1.0585	0.0249	0.1272
PAS	0.9981	0.979	1.0175	-0.0019	0.8449
Rappel des prises médicamenteuses par les parents	1.5931	0.7538	3.3665	0.4657	0.2226
Bonne connaissance du traitement	0.3594	0.155	0.8331	-1.0234	0.017
Connaissance de la gravité de l'HTA	0.3445	0.1264	0.939	-1.0655	0.0373

## Discussion

L'observance médicamenteuse occupe une place de choix parmi les causes de non contrôle tensionnel [2, 3]. Chez des patients hypertendus traités, les taux de mauvaise observance sont très variables, allant de 30 à 80% selon les études [8, 9]. Cette variabilité des données est liée entre autres à la différence des méthodes de mesure, d’échantillonnage et de durée de suivi. Le taux de 32.5% rapporté dans ce travail, proche de celui de Schmitt aux USA de 33% [10], est nettement moins important que ceux rapportés par Konin en Côte d'Ivoire [11] et Ghozzi en Tunisie [12], 55% et 63.4% respectivement. Cependant, Girerd et al [13] en France ont rapporté un meilleur résultat (8%) dans une population d'hypertendus suivis dans un milieu hospitalier spécialisé.

Les facteurs de mauvaise observance sont de deux ordres: ceux liés au patient et ceux propres à la maladie elle-même (HTA). Concernant les facteurs liés au patient, notre étude a montré que les patients non observants étaient en moyenne plus âgés que les autres. Dans la littérature, le rôle joué par l’âge dans l'observance est controversé. Ainsi, dans certaines études [6, 13, 14], il apparaît que les sujets plus jeunes étaient moins observants, alors que dans d'autres [10, 15, 16, 17] il n'a pas été retrouvé de relation entre l’âge et l'observance médicamenteuse chez les patients hypertendus. D'autres auteurs [18] avaient objectivé une moins bonne observance chez les sujets âgés, s'expliquant en partie par l'existence des troubles cognitifs très fréquents à cette période de la vie. Globalement, et comme dans notre étude, aucune relation n'est établie entre le sexe et le niveau d'observance, avec des résultats souvent contradictoires entre différentes études [11–14, 19, 20].

En l'absence de couverture sociale dans l'immense majorité des cas, nous avons montré que la perception du coût élevé du traitement, ainsi que l'achat des médicaments par une tiers personne étaient liés à une mauvaise observance. Pour Adoubi et al [5] en Côte d'Ivoire, l'absence d'une assurance maladie était un facteur de risque de mauvaise observance, dans une étude où près de la moitié des sujets (49.4%) bénéficiaient d'une couverture sociale. Dans notre étude, certains facteurs tels que la connaissance de son traitement et des complications de l'HTA ainsi que la perception de la gravité de l'HTA étaient fortement liés à une bonne observance. Girerd et al [13] ont également mis en évidence la relation entre la mauvaise observance et une mauvaise connaissance de son traitement antihypertenseur. L'appropriation du traitement par le patient est donc un élément important sur lequel les prescripteurs devraient insister, d'où la place fondamentale de l’éducation thérapeutique chez ces patients atteints de pathologies chroniques, comme le recommande l'OMS [21]. Plusieurs programmes d’éducation des patients sur la maladie et les objectifs du traitement ont démontré leur influence sur l'amélioration de l'observance surtout lorsque ceux-ci intègrent les concepts de sociologie et de mode de vie [22].

Concernant les facteurs liés à l'HTA, nous n'avons pas retrouvé de relation entre le contrôle tensionnel et le niveau d'observance, tout comme Adoubi et al en Côte d'Ivoire [5]. Pourtant, cette relation a été établie dans certaines études [12, 13, 23], mettant en exergue le fait que les non répondants au traitement ne sont pas toujours des non observants [16]. Nous n'avons pas retrouvé dans ce travail, une relation entre le niveau d'observance et la présence d'une co-morbidité. Pour certains auteurs [24], la présence d'un diabète sucré ou d'une dyslipidémie améliorait l'observance; alors qu'inversement, la dépression était associée à une mauvaise observance.

La présence d'une complication de l'HTA était identifiée dans notre travail ainsi que dans celui d'Adoubi et al [5], comme facteur de risque de mauvaise observance, objectivant l'effet néfaste de la mauvaise observance sur le pronostic des patients hypertendus.

## Conclusion

Cette étude préliminaire a révélé le faible niveau d'observance médicamenteuse chez des patients hypertendus congolais. La mauvaise connaissance du traitement et l'ignorance de la gravité de l'HTA ont été les principaux facteurs prédictifs de mauvaise observance. Ainsi, pour améliorer l'observance de nos patients, afin de contribuer à la réduction de l'incidence des complications et des coûts liés à la prise en charge de l'HTA, il importe d'insérer dans l'arsenal thérapeutique des patients hypertendus, des programmes d’éducation thérapeutique.
